# Data on trend changes of drinking groundwater resources quality in Sabzevar city (A case study)

**DOI:** 10.1016/j.dib.2018.08.175

**Published:** 2018-09-01

**Authors:** Sima Zamand, Hossein Alidadi, Aliasghar Najafpoor, Vahid Taghavimanesh, Nooshin Akbari Sharak, Hossein Najafi Saleh

**Affiliations:** a Department of Environmental Health Engineering, School of Health, Mashhad University of Medical Sciences, Mashhad, Iran; b Student Research Committee, Department of Biostatistics and Epidemiology, School of Public Health, Mashhad University of Medical Sciences, Mashhad, Iran; c Department of Environmental Health Engineering, Torbat Heydarieh University of Medical Sciences, Torbat Heydarieh, Iran

**Keywords:** Trend, Groundwater, Sabzevar

## Abstract

The data of this study was conducted to evaluate the groundwater quality trend changes in Sabzevar (Iran, Khorasan Razavi) during one decade (2006–2016). The Mean ± SD of electrical conductivity (as µmhos/cm), total hardness (as calcium carbonate, mg/l) and total dissolved solid in the first and end year of the study were 605.45 ± 194.69 and 695.22 ± 288.52, 198.77 ± 56.83 and 214.45 ± 84.73, 350.25 ± 106.81 and 486.44 ± 183.52 respectively. At the end of the results were compared with WHO (World Health Organization) guideline and Iranian drinking water standard (No. 1053). The results show that all of the evaluated parameters were less than the WHO guideline and the Iranian drinking water standard, only the TH (Total Hardness) was higher than the standard range. On the basis of Pearson correlation coefficient, the ascending trend of some parameters concentration with time was significant at the level of 95% of confidence limits (*α* ≤ 0.05).

**Specifications Table**

**Table:** 

Subject area	Water chemistry
More specific	Describe narrower subject area
Type of Data	Tables, Figures
How data was acquired	The required data were collected from the results recorded in the water in the Water and Wastewater Company of Khorasan Razavi province during the years 2006–2016
Data format	Raw, Analyzed
Experimental factors	All water samples in polyethylene bottles were stored in a dark place at room temperature until the parameters analysis
Data source location	Sabzevar, Khorasan Razavi province, Iran
Data accessibility	Data are included in this article and supplement file excel

**Value of data**

• Determination of the physical and chemical parameters including pH, TDS, TH, EC, Ca^2+^, Mg^2+^, ${\mathrm{HCO}}_{3}^{-}$ , Na^+^, K^+^, Cl^−^ and ${\mathrm{SO}}_{4}^{2-}$ in groundwater was evaluated in Sabzevar urban area, Khorasan Razavi province, Iran.
• Continuing the ascending trend of the parameters concentration and declining the quality of water resources and incompatibility with Iranian drinking water standard can lead to significant health risks.
• Data of this study can help to better understand the quality of groundwater in this area.
• Tracking the trend changes, investigating the reasons and preventive measures are important.

## 1 Data

Data presented here deal with monitoring of physical and chemical including pH, Na^+^, Ca^2+^, Mg^2+^, K^+^, EC, TDS, HCO^3−^, ${\mathrm{SO}}_{4}^{2-}$, Cl^−^, and TH as in Sabzevar, Khorasan Razavi Province, Iran. Fig. 1 shows the study area. A summary of Water quality characteristics and correlation of the parameters with time are presented in Table 1, Table 2 respectively. Charts 1–11 show trend changes in parameters in the years (2006–2016).

**Fig. 1:**
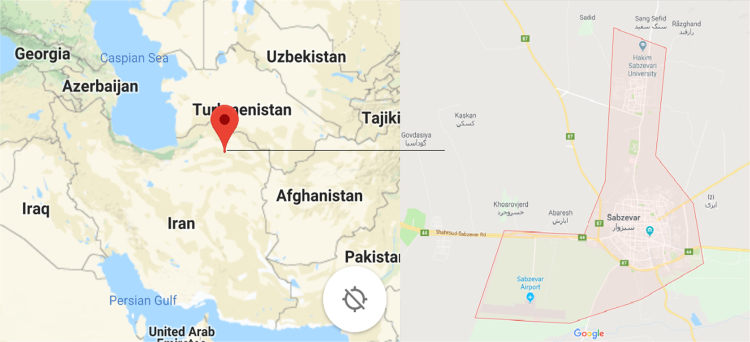
Location of the study area in Sabzevar, Khorasan Razavi province, Iran.

**Table 1:** Chemical analysis report of water quality of drinking groundwater resource of Sabzevar city.

**Year**	**pH**	**Na**^**+**^ (mg/L)	**Ca**^**2+**^ (mg/L)	**Mg**^**2+**^ (mg/L)	**K**^**+**^ (mg/L)	$Hco_{3}^{-}$ (mg/L)	$So_{4}^{2-}$ (mg/L)	**Cl**^**−**^ (mg/L)	**TDS** (mg/L)	**TH** (mg/L as CaCO_3_)	**EC (**µmhos/cm)
2006	7.25	50.85	43.15	20.08	1.22	218.20	73.40	25.75	350.25	198.77	605.45
2007	8.04	50.95	44.25	22.25	1.15	250.36	94.48	26.45	375.05	201.23	625.24
2008	8.49	48.66	49.78	19.85	3.04	203.45	82.24	28.12	401.25	205.05	614.12
2009	8.44	49.56	52.32	24.22	1.46	247.25	98.17	23.55	425.13	210.22	536.21
2010	7.82	49.55	52.65	23.25	1.38	245.55	101.25	26.02	385.25	215.99	715.12
2011	7.58	51.60	54.56	22.62	1.33	232.25	95.25	21.32	437.52	228.45	725.23
2012	8.09	56.38	59.45	26.04	2.25	262.81	91.17	24.74	445.02	210.12	725.45
2013	7.41	53.39	55.31	23.12	1.56	259.25	96.25	30.54	450.35	219.45	733.12
2014	7.33	52.22	54.38	24.38	2.22	248.55	91.78	26.38	456.25	233.66	712.22
2015	7.20	49.52	47.22	23.12	1.59	212.45	99.74	27.09	407.85	224.76	732.12
2016	7.33	50.45	49.64	22.97	1.23	262.25	85.45	28.13	486.44	214.45	695.22
Mean	7.72	51.19	51.15	22.90	1.67	240.21	91.74	26.19	420.03	214.74	674.50
Max	8.49	56.38	59.45	26.04	3.04	262.81	101.25	30.54	486.44	233.66	733.12
Min	7.20	48.66	43.15	19.85	1.15	203.45	73.40	21.32	350.25	198.77	536.21
S.D	0.476	2.196	4.930	1.782	0.586	20.686	8.381	2.456	40.250	11.150	67.373
**WHO**	6.5–8.5	400	250	150	**–**	**–**	200	200	500	200	**–**
**1053IR**	6.5–8.5	200	300	30	**–**	**–**	250	250	1000	200	**–**

**Table 2:** Pearson correlation of the chemical parameters.

	**pH**	**Na**^**+**^	**Ca**^**2+**^	**Mg**^**2+**^	**K**^**+**^	$Hco_{3}^{-}$	$So_{4}^{2-}$	**Cl**^**−**^	**TDS**	**TH**	**EC**
**pH**	1										
**Na**^**+**^	−.121	1									
**Ca**^**2+**^	−.575	.465	1								
**Mg**^**2+**^	−.470	.137	.672*	1							
**K**^**+**^	−.168	.388	.444	.590	1						
$Hco_{3}^{-}$	−.226	−.054	.177	−.113	.112	1					
$So_{4}^{2-}$	.099	.018	.345	.519	.210	.018	1				
**Cl**^**−**^	−.030	.599	.252	.128	.568	.599	.252	1			
**TDS**	.434	.100	.010	.052	.148	.100	.010	.052	1		
**TH**	.020	.610*	.392	.456	.577	.610*	.392	.456	.577	1	
**EC**	.177	.629*	.475	.511	.658*	.629*	.475	.511	.658*	−.228	1

* Correlation is significant at the 0.05 level (2-tailed)

## 2 Experimental design, materials and methods

### 2.1 Study area description

Sabzevar is one of the largest cities in Khorasan Razavi province in Northeastern Iran. Sabzevar is located in Khorasan Razavi province at UTM coordinates of *X* = 560516.13 and *Y* = 4011898.18 (57°40′ East longitude and 36°15′ North latitude) [1], [2], [3], [4], [5]. The climate of the Sabzevar is dry, and the precipitation average is 188.6 mm/year. Also the air׳s highest and lowest temperatures are 42.5 °C and −5.1 °C, respectively, with an annual average of 18.5 °C (Fig. 1).

### 2.2 Data collection

The required data were collected from the results recorded in the water in the Iran Water resources management Company during the years 2006–2016. In this study, 805 samples were analyzed by descriptive and analytical statistics (correlation coefficients) in 10 years (Table 3). The important major cations and anions in water samples were analyzed following a standard method (APHA 2008) [6], [7], [8], [9], [10], [11], [12], [13], [14], [15], [16].

**Table 3:** Number of water samples in years studied (2006–2016).

**Year**	**Number of samples**
2006	68
2007	56
2008	85
2009	80
2010	65
2011	75
2012	73
2013	84
2014	79
2015	72
2016	68
**Total**	**805**
