# Supplementary material for: Data on trend changes of drinking groundwater resources quality in Sabzevar city (A case study)
Source: Data Brief. 2018 Sep 1;20:889–93. doi: 10.1016/j.dib.2018.08.175 (PMC6138976; doi:10.1016/j.dib.2018.08.175)
Supplement: Supplementary file 1 — Supplementary material [file mmc1.docx]

None declare
